# Lipids Metabolism Inhibition Antiproliferative Synergy with 5-Fluorouracil in Human Colorectal Cancer Model

**DOI:** 10.3390/ijms26031186

**Published:** 2025-01-30

**Authors:** Judyta Zabielska, Ewa Stelmanska, Sylwia Szrok-Jurga, Jarosław Kobiela, Aleksandra Czumaj

**Affiliations:** 1 Department of Biochemistry, Faculty of Medicine, Medical University of Gdansk, 80-211 Gdansk, Poland; judyta.zabielska@gumed.edu.pl (J.Z.); ewa.stelmanska@gumed.edu.pl (E.S.); szrok@gumed.edu.pl (S.S.-J.); 2 Department of Surgical Oncology, Transplant Surgery and General Surgery, Faculty of Medicine, Medical University of Gdansk, 80-211 Gdansk, Poland; jaroslaw.kobiela@gumed.edu.pl; 3 Department of Pharmaceutical Biochemistry, Faculty of Pharmacy, Medical University of Gdansk, 80-211 Gdansk, Poland

**Keywords:** CRC cells, colorectal cancer, lipid metabolism, cell viability, combination therapy, avasimibe

## Abstract

Colorectal cancer (CRC) is recognized as the third most lethal cancer worldwide. While existing treatment options demonstrate considerable efficacy, they are often constrained by non-selectivity and substantial side effects. Recent studies indicate that lipid metabolism significantly influences carcinogenesis, highlighting it as a promising avenue for developing targeted anticancer therapies. The purpose of the study was to see if acyl-coenzyme A: cholesterol acyltransferase 1 (ACAT1), 3-hydroxy-3-methylglutaryl-CoA reductase (HMGCR), and stearoyl-CoA 9-desaturase (SCD1) are good metabolic targets and whether the use of inhibitors of these enzymes together with 5-fluorouracil (5-FU) would have a synergistic effect on CRC cell viability. To confirm that the correct lipid targets were chosen, the expression levels of ACAT1, HMGCR, and SCD1 were examined in CRC patients and cell models. At first, each compound (Avasimibe, Lovastatin, MF-438, and 5-FU was tested separately, and then each inhibitor was paired with 5-FU to assess the synergistic effect on cell viability. Gene expression of selected enzymes significantly increased in tissue samples obtained from CRC patients and cancer cell lines (HT-29). Inhibition of any of the selected enzymes reduced CRC cell growth in a dose-dependent manner. More importantly, the combination of 5-FU + Avasimibe (an ACAT1 inhibitor) and 5-FU + MF-438 (an SCD1 inhibitor) produced a stronger antiproliferative effect than the inhibitors alone. 5-FU combined either with Avasimibe or MF-438 showed a synergistic effect with an HSA score of 47.00 at a dose of 0.3 + 30 µM, respectively (2.66% viability rate vs. 46%; *p* < 0.001), and 39.34 at a dose of 0.3 + 0.06 µM (46% vs. 10.33%; *p* < 0.001), respectively. The association of 5-FU with Lovastatin (HMGCR inhibitor) did not significantly impact CRC cell viability in a synergistic manner. Inhibition of lipid metabolism combined with standard chemotherapy is a promising strategy that reduces CRC cell viability and allows for the use of a lower drug dose. The combination of 5-FU and Avasimibe has the greatest therapeutic potential among studied compounds.

## 1. Introduction

The death rate for colorectal cancer (CRC) is 9.4%, rendering it the third most lethal cancer worldwide [1]. The estimates suggest that fewer than 20% of patients with metastatic CRC survive beyond five years post-diagnosis [2]. Surgery, radiotherapy, and cytotoxic chemotherapy based on 5-fluorouracil (5-FU), irinotecan, cetuximab, and oxaliplatin constitute the primary methods of treatment [3,4]. For unresectable metastatic CRC, the preferred treatment options include chemotherapy, biological therapy such as antibodies targeting cellular growth factors, immunotherapy, or a combination of these approaches [2,5]. However, their effectiveness is limited [3].

**Figure 1:**
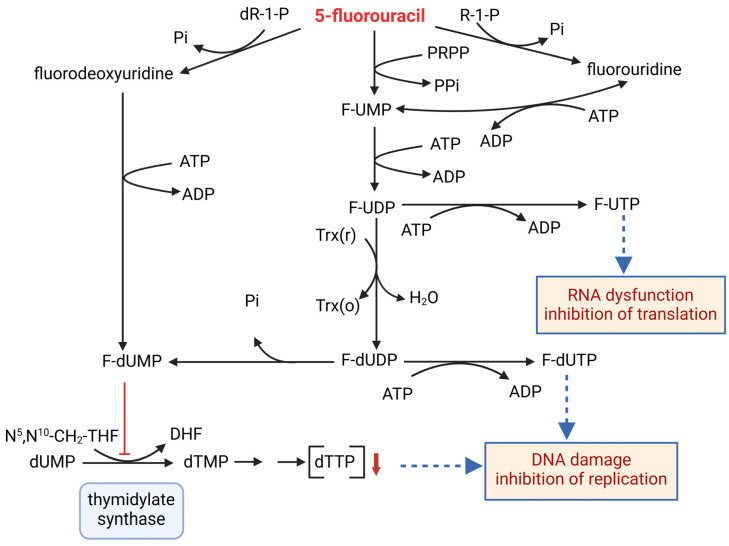
Molecular consequences of administration of 5-fluorouracil. R-1-P—ribose monophosphate; dR-1-P—deoxyribose monophosphate; Pi—phosphate; PRPP—5′-phosphoribosyl-1-pyrophosphate; PPi—pyrophosphate; F-UMP—fluorouridine monophosphate; F-UDP—fluorouridine diphosphate; F-UTP—fluorouridine triphosphate; F-dUMP—fluorodeoxyuridine monophosphate; F-dUDP—fluorodeoxyuridine diphosphate; F-dUTP—fluorodeoxyuridine triphosphate; dUMP—deoxyuridine monophosphate; dTMP—deoxythymidine monophosphate; dTTP—deoxythymidine triphosphate; 5,10-CH_2_-THF—N5,N10 Methylenetetrahydrofolate; DHF—dihydrofolate; Trx(r)—thioredoxin reduced; Trx(o)—thioredoxin oxidized; ATP—adenosine triphosphate; ADP—adenosine diphosphate; DNA—deoxyribonucleic acid; RNA—ribonucleic acid.

5-fluorouracil (5-FU) is an antimetabolite used as first-line chemotherapy to treat various types of cancer, including colorectal cancer [6]. It can be administered intravenously or orally in the form of the precursor capecitabine [7]. 5-FU enters cells via the same transport mechanism as uracil, where it is rapidly converted into fluorouridine, fluorodeoxyuridine, and fluorouridine monophosphate (FUMP). Their metabolism then results in the formation of active metabolites, such as fluorodeoxyuridine monophosphate (F-dUMP) and fluorouridine triphosphate (F-UTP), causing cytotoxic effects through two main mechanisms: inhibition of replication and translation [8]. The transformations of these antimetabolites in the cell are demonstrated in Figure 1. F-dUMP is an irreversible inhibitor of thymidylate synthase, which is involved in the synthesis pathway of dTTP, one of the substrates of DNA polymerase. F-dUMP thus leads to an imbalance of deoxynucleotide triphosphates (dNTPs), resulting in inhibition of DNA replication and cell death. 5-FUTP exerts its cytotoxic effects by attaching to RNA, leading to inhibition of translation. Moreover, the accumulation of deoxyuridine triphosphate (dUTP) and fluorodeoxyuridine triphosphate (F-dUTP) leads to the incorrect incorporation of uracil into DNA. Uracil bases are removed by uracil DNA glycosylase, generating apyrimidinic sites. In the final, a futile cycle of misincorporation and misrepair results in DNA strand breaks and permanent damage [9].

Lipid metabolism plays a significant role in tumorigenesis, with several mechanisms contributing to its dysregulation in cancer cells [10,11]. Notably, cancer cells frequently exhibit increased demands for lipids to support their rapid proliferation and growth. The focus is primarily on fatty acids (FAs) and cholesterol, which are crucial components of biological membranes. Furthermore, FAs are essential not only for the synthesis of membrane phospholipids but also serve as an energy source for tumor cells [12]. Research indicates that the uptake of free fatty acids (FFAs) from adipocytes surrounding cancer cells enhances fatty acid oxidation (FAO) in colorectal cancer [13]. Additionally, it was demonstrated that elevated FAO may facilitate cancer metastasis by increasing ATP levels, preventing cancer cells from apoptosis, and stimulating the epithelial–mesenchymal transition [14]. FFAs are also an essential substrate for the synthesis of compounds stored in lipid droplets: triacylglycerols (TAGs) and cholesterol esters (CEs). It has been demonstrated that lipid storage is also important for cancer cell growth and metastasis. Therefore, enzymes implicated in lipid synthesis and storage represent potential targets for anticancer pharmacotherapy. Moreover, modified lipid metabolism co-regulates signalling pathways associated with cell survival [10,11]. Lipid mediators, including diacylglycerol, phosphatidic acid, and lysophosphatidic acid, which are partially composed of FAs, may promote tumorigenesis, including proliferation, migration, invasion, and angiogenesis [15]. Additionally, the development of cancer cells is significantly influenced by cholesterol metabolism, which is not limited to its role in the construction of biological membranes. It was demonstrated that cholesterol metabolites, including oxysterols, exhibit regulatory effects on the proliferation of cancer cells [16]. Moreover, elevated cholesterol levels in tumor cells facilitate their escape from immune surveillance [16]. The significant impact of lipid metabolism on cancer cell proliferation has prompted investigations into tumor suppression through the inhibition of lipid metabolism pathways.

**Figure 2:**
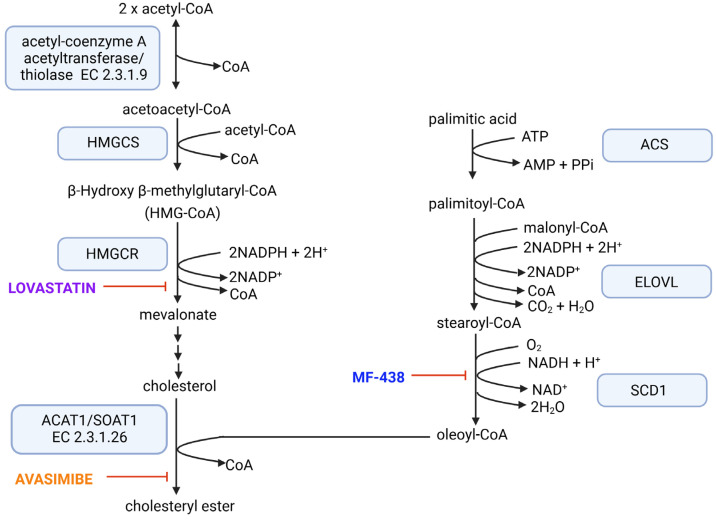
Molecular targets of used inhibitors. ACS—Acyl-CoA synthetases, ELOVL—fatty acid elongases, HMGCR—hydroxymethylglutaryl-CoA reductase, HMGCS—hydroxymethylglutaryl-CoA synthase, SCD1—stearoyl-CoA desaturase-1. In the case of enzymes for which the same abbreviation is sometimes used, the EC number is also provided.

A distinct antiproliferative effect has been demonstrated through the inhibition of the cholesterol synthesis pathway, specifically targeting 3-hydroxy-3-methylglutaryl-CoA reductase (HMGCR), the rate-limiting enzyme in this pathway [17,18,19], as well as cholesterol storage by inhibiting acyl-coenzyme A: cholesterol acyltransferase (ACAT) [20,21]. There are two isoforms of ACAT in mammalian cells [21]. ACAT1 is the major isoenzyme expressed in all examined tissues, including cancer cells, while ACAT2 is mainly localized in lipoprotein-secreting cells (enterocytes and hepatocytes) [22]. The reaction catalyzed by ACATs is the conversion of cholesterol to cholesteryl esters (see Figure 2). Among long-chain fatty acids, oleic acid (18:1) is the preferred substrate for ACAT1 [21]. Several studies demonstrated the effect of inhibiting enzymes associated with FA synthesis (particularly FASN and SCD1 [23,24,25,26], as well as FA storage [16]) on the proliferation of cancer cells.

The subject of our scientific research is lipid metabolism in colorectal cancer (CRC). This cancer is one of the leading causes of mortality among oncology patients; therefore, the search for new therapies is still ongoing. Deregulation of lipid metabolism is increasingly recognized as a potential target for cancer treatment; however, evidence supporting its role as a diagnostic and prognostic biomarker in CRC remains insufficient. In general, the expression of genes associated with lipid synthesis increases in tumors; therefore, we aimed to examine the in vitro effects of inhibitors of significant enzymes involved in cholesterol metabolism (HMGCR and ACAT1) and monounsaturated FA synthesis (SCD1) in a CRC cell model. The metabolic linkages of the reactions catalyzed by these enzymes are shown in Figure 2. Moreover, we analyzed the mRNA levels of these enzymes in samples obtained from patients diagnosed with CRC. In a previous study, we demonstrated that inhibition of FASN activity with orlistat leads to reduced proliferation in CRC cells [24]. This time, we aimed to determine whether simultaneous administration of inhibitors of enzymes involved in lipid metabolism combined with 5-FU could induce a stronger antiproliferative effect on CRC cells. Investigating the effect of lipid metabolism inhibitors in conjunction with 5-FU in a CRC model aims to identify the most potent combination to reduce the dosage and side effects of chemotherapy used separately.

## 2. Results

### 2.1. Gene Expression of Selected Targets

**Figure 3:**
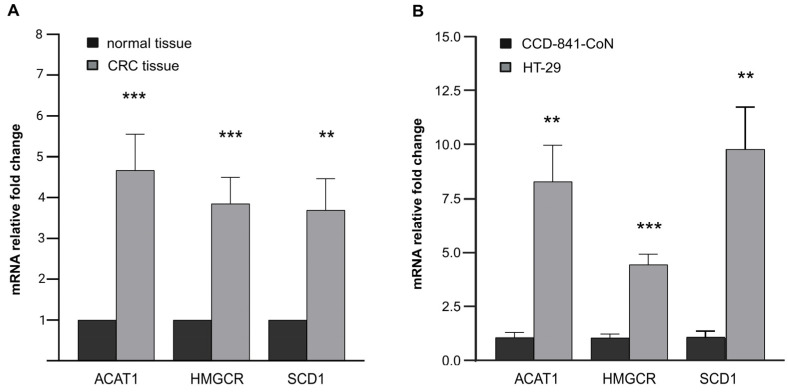
ACAT1, HMGCR, and SCD1 mRNA levels (**A**) in colon cancer tissue samples from CRC patients vs. normal colon mucosa tissues obtained from CRC patients; (**B**) in HT-29 cells colon cancer cell line vs. CCD-841-CoN control colon cell line. Data are presented as mean ± SEM. Statistical significance in comparison to the control group was marked as follows: for ** *p* ≤ 0.01; *** *p* ≤ 0.001.

The mRNA levels of ACAT1, HMGCR, and SCD1 in CRC tissue samples from patients were significantly higher in comparison to normal intestinal mucosa tissue of the same subject (Figure 3A). Similarly, compared to the normal colon cell line CCD-841-CoN, the CRC cell line HT-29 showed significantly elevated expression of ACAT1, HMGCR, and SCD1 genes (Figure 3B). The evaluation of the mRNA level of these genes in both the patient’s tissue and cell line confirms the selection of an appropriate in vitro model.

### 2.2. Treatment with a Single Experimental Agent

**Figure 4:**
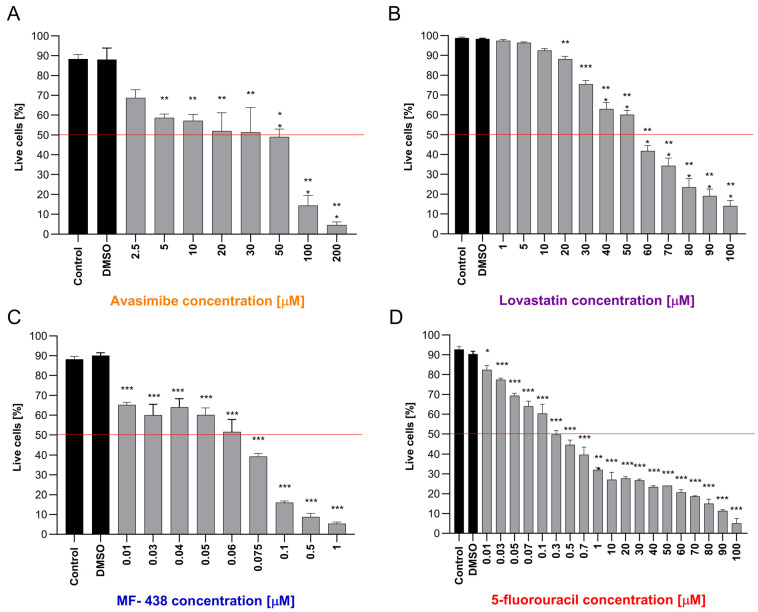
The effect of treatment with increasing concentrations of (**A**) Avasimibe, (**B**) Lovastatin, (**C**) MF-438, and (**D**) 5-fluorouracil (μM) on HT-29 cell viability. The red line represents the IC50 cut-off. Data are shown as mean ± SEM. Statistical significance in comparison to the control group is marked as follows: for * *p* ≤ 0.05; ** *p* ≤ 0.01; *** *p* ≤ 0.001.

Treatment of the HT-29 cell line with increasing concentrations of selected experimental agents (Avasimibe, Lovastatin, MF-438, and 5-FU) led to progressive inhibition of cell viability (Figure 4A–D).

**Figure 5:**
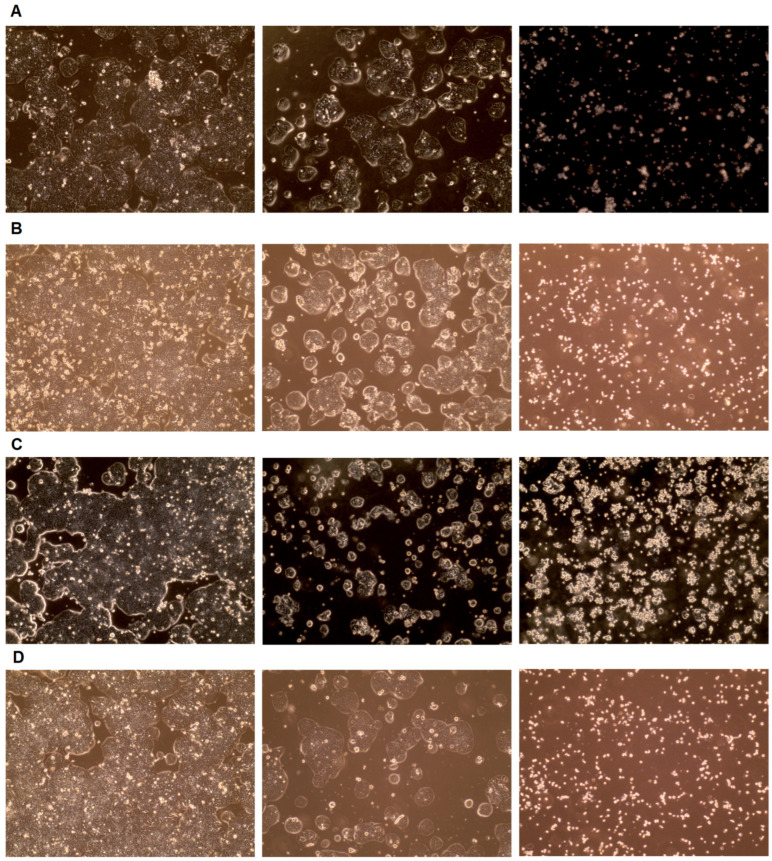
Cell morphology after treatment with (**A**) Avasimibe, (**B**) Lovastatin, (**C**) MF-438, and (**D**) 5-fluorouracil (5-FU). Random representations of approximately IC50 and the highest tested concentration were selected.

Each agent was characterized by its specific concentration at which it achieved IC50. A 50% inhibition of HT-29 cell viability occurred after treatment with 30 μM Avasimibe, 55 μM Lovastatin; 0.06μM MF-438, and 0.3 μM 5-FU. Each tested inhibitor (Avasimibe, Lovastatin, and MF-438) limited CRC cell viability, which allows them to be considered promising anticancer agents. Moreover, for each of the factors tested, the IC 50 for CRC cells was lower than the IC50 for normal colon cells (Supplementary Figure S1). One of the tested inhibitors, MF-438, achieved IC50 at a lower dose than the chemotherapeutic agent, 5-FU. No differences were observed between control conditions and DMSO treatment (solvent control). This proves that any observed changes were not related to the presence of this solvent in the cell cultures. Based on microscope images, HT-29 cell line growth and number were assessed (Figure 5). As the concentration of each experimental agent increased, fewer cells were observed. The morphology of the cells has also changed. The cells underwent a size reduction and lost their characteristic shape.

### 2.3. Treatment with the Combination of Inhibitors and 5-FU

**Figure 6:**
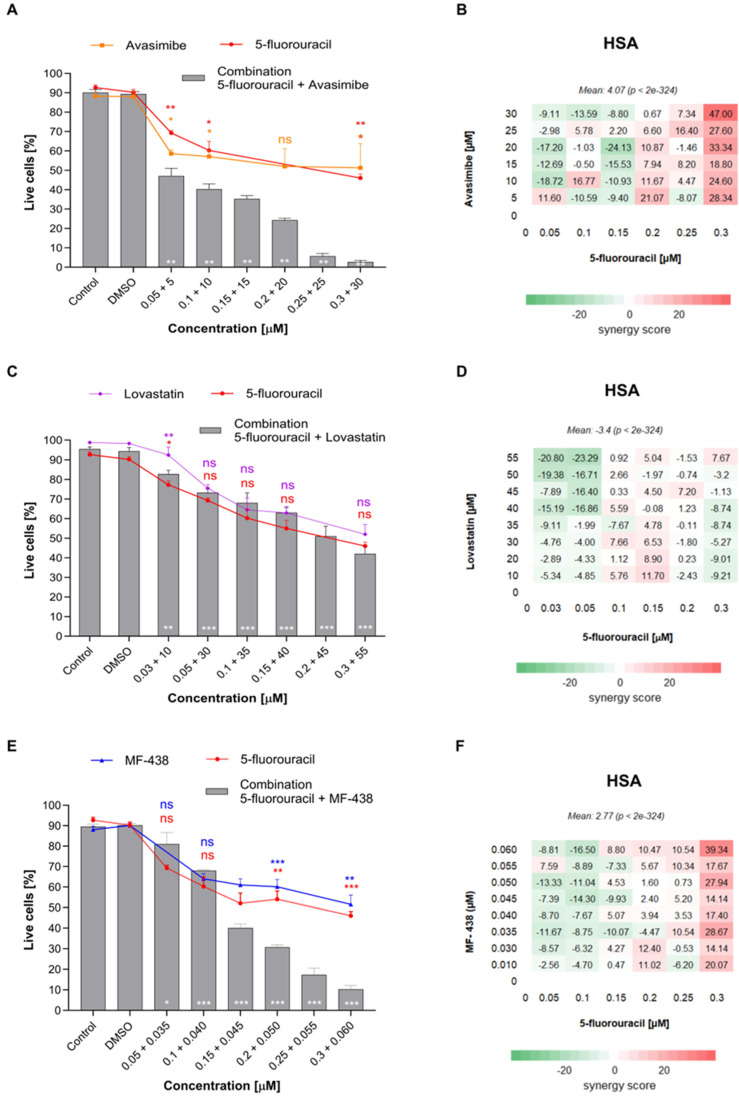
The effect cell viability and synergy heat map of treatment with increasing concentrations of (**A**,**B**) 5-FU and Avasimibe, (**C**,**D**) 5-FU and Lovastatin, and (**E**,**F**) 5-FU and MF-438. Data on graphs are shown as mean ± SEM. Statistical significance in comparison to the control group is marked down on the representing bars in white; whereas comparison to the result of drug effect alone is marked on the top of the bar in the corresponding color as follows: for * *p* ≤ 0.05; ** *p* ≤ 0.01; *** *p* ≤ 0.001, ns no statistical significance. On heat maps, each square represents a synergy score for a specific dose of drug combination.

To assess potential synergistic effects on cell viability, each inhibitor was paired with a 5-FU. Treatment of HT-29 cells with combinations of two agents significantly decreased the cell viability (Figure 6A–F). A 50% inhibition of HT-29 cell viability occurred after treatment with 0.05 + 5 µM 5-FU and Avasimibe (Figure 6A), 0.3 + 55 µM 5-FU and Lovastatin (Figure 6C), and 0.15 + 0.045 µM 5-FU and MF-438 (Figure 6E). The 5-FU, when combined with Avasimibe, showed a stronger inhibiting effect on CRC cell viability than acting alone. The additive effect was observed even at the smallest doses, whereas high doses were shown to have a greater synergic effect (Figure 6B). A mixture of 0.3 + 30 µM resulted in only 2.66% live cells left compared to 46% when 5-FU acted alone (*p* < 0.001). A high HSA synergy score of 47.00 was shown for this dose. The overall HSA synergy score for this combination was 4.07 and was the highest of all the combinations tested. This suggests that this combination is the most promising. The 5-FU combined with Lovastatin did not show a stronger inhibiting effect on CRC cells in comparison to acting alone (Figure 6D). Some decrease in live cell number was observed for small doses like 0.03 + 10 µM, where the percentage of live cells decreased from 92.44 for Lovastatin to 82.67 for the combination (*p* < 0.01) but increased in comparison to the 77.33% obtained by 5-FU alone (*p* < 0.05). The overall HSA synergy score for this combination was −3,40 and was the lowest of all the combinations tested. The combination of 5-FU and MF-438 was shown to be more potent in CRC cell viability inhibition in higher doses (Figure 6F). Cell viability was decreased from 46% to 10.33% in 0.3 + 0.06 µM mixture (*p* < 0.001) and from 60.09% to 30.67% in 0.2 + 0.05 µM dose (*p* < 0.001). The highest synergy score of 39.34 was demonstrated for dose 0.3 + 0.06 µM. The overall HSA synergy score for this combination was 2.77. This suggests that, in general, these two compounds have some synergistic effect in a few select high concentrations, but the effect is weaker than the combination of 5-FU and Avasimibe. To confirm the observed synergy, a combination index (CI) was also calculated (Supplementary Table S1). The microscope images from combinations of 5-FU and inhibitors are available in Supplementary Figure S2.

## 3. Discussion

The administration of 5-FU is linked to various potential side effects, exhibiting a spectrum of severity. The most commonly reported adverse effects include nausea, vomiting, diarrhea, loss of appetite, fatigue, weakness, mouth sores, hair loss, and skin reactions [27]. Additionally, particular individuals might experience less prevalent complications, such as dizziness, a metallic taste in the mouth, and photosensitivity, which may elevate the risk of sunburn. Negative consequences that require immediate medical attention include unusual bleeding or bruising, chest pain or shortness of breath, and signs of infection, such as fever, chills, or blood in urine or stools [28]. Some patients have been observed to have severe allergic reactions, which may present as difficulty breathing, swelling of the face or throat and hives, heart problems, such as arrhythmia or myocardial infarction [29], and neurological symptoms, including confusion or trouble with balance [30]. Furthermore, resistance to 5-FU remains the primary challenge that impacts the efficacy of chemotherapy in CRC [12]. Considering this alongside low survivability necessitates the development of new approaches.

Targeting lipid metabolism has emerged as a promising strategy in the field of cancer therapy. Despite the potential benefits, lipid metabolism inhibitors have not yet received approval for use in cancer treatment. Ongoing research is focused on developing innovative treatment concepts that leverage alterations in lipid metabolism in conjunction with other anticancer modalities. In the present study, we provide preliminary documentation of the ability of selected lipid metabolism inhibitors to inhibit cancer cell proliferation in an in vitro model. Furthermore, we investigate the potential of these inhibitors to enhance the sensitivity of CRC cells to chemotherapeutic agents (5-FU) when utilized in combination.

ACAT1 is frequently overexpressed in various types of cancer, including ovarian, prostate, pancreatic, clear cell renal cell carcinoma, and CRC, which aligns with our findings [31,32,33]. Studies have shown that higher levels of ACAT are associated with more aggressive tumor behavior and poorer patient outcomes, highlighting its potential as a therapeutic target. In a colorectal cancer model, toll-like receptor 4 (TLR4) was shown to promote cell proliferation, migration, and invasion by increasing ACAT1 expression in HT29 [34]. Avasimibe, an ACATs inhibitor, has been shown to enhance antitumor effects when used in combination with other therapeutic agents. For example, it has been found to increase chemosensitivity to cisplatin in human primary ovarian epithelial cells (H-6036) [31]. Additionally, a combination of Avasimibe and the anti-PD-1 antibody (anti-programmed cell death protein 1 antibody) leads to improved efficacy compared to monotherapy in inhibiting melanoma progression [35]. Furthermore, Avasimibe combined with nanoparticles of doxorubicin (an anticancer drug widely used in many cancers to induce tumor cell apoptosis) has demonstrated enhanced anti-tumor efficacy in models of breast cancer progression [36]. To our knowledge, this study represents the first investigation into the synergistic effects of Avasimibe and 5-FU. By combining 5-FU with Avasimibe, we were able to successfully decrease the effective dose of 5-FU by six times. This reduction, when applied therapeutically to patients, could lead to a significant decrease in 5-FU-induced side effects. Nevertheless, it should be remembered that the use of Avasimibe may also be associated with side effects. However, preclinical studies on beagle dogs have shown significant toxicity restricted to a ≥300 mg/kg dose [37]. The mechanism of the synergistic effect of 5-FU and Avasimibe on the inhibition of CRC cell proliferation is unknown. Presumably, the underlying mechanism may be the effect of free cholesterol on regulating various gene expressions. Briefly, ACAT inhibition leads to an increase in free cholesterol accumulation in ER membranes. This leads to inhibition of the activation of the transcription factor SREBP2, which affects the expression of many genes, mainly related to cholesterol metabolism, but not only. Among other things, SREBP2 has been shown to regulate the expression of the caveolin-1 gene, which participates in tumorigenesis through the caveolin-1/MAPK pathway. Cholesterol not only has structural functions in membranes but also leads to changes in the conformation of membrane proteins involved in intracellular signaling activity, which may be responsible for the dysregulation of signaling. Associations between SREBP2 and PI3K/AKT/MTOR, MAPK, AR, and p53 have been demonstrated [38]. Cholesterol cytotoxicity may also be associated with the induction of ER stress, resulting in apoptosis. Significantly elevated levels of ER stress markers such as GRP78 (78 kDa glucose-regulated protein), ATF4 (activating transcription factor 4), and CHOP (homologous protein C/EBP) were observed in colorectal cancer cells treated with ACAT inhibitor [39]. In conclusion, the simultaneous application of 5FU and Avasimibe leads to the inhibition of key processes in dividing cells: translation, replication, as well as changes in intracellular signaling and induction of ER stress.

We did not find any synergy effect by combining Lovastatin with 5-FU. However, a few reports have demonstrated that Lovastatin enhances the chemosensitivity of CRC cells to 5-FU [40,41]. The discrepancy might be explained due to the different treatment set-up. In the cited studies, the CRC cell lines were first incubated with Lovastatin and then with 5-FU, whereas in this study, the CRC cell line was at the same time incubated with both Lovastatin and 5-FU. Moreover, phase II clinical studies have shown that high-dose Lovastatin does not appear to be effective for patients with advanced gastric adenocarcinoma [42].

In preclinical studies, inhibiting SCD1 with MF-438 has been shown to reverse resistance to cisplatin in non-small lung cancer stem cell lines and increase the effectiveness of radiation therapy in esophageal squamous cell carcinoma cell lines [43,44]. In our study, we presented that MF-438 can also increase the effectiveness of the 5-FU in the CRC model. We successfully reduced the dose of 5-FU by half while maintaining the same effect on cell viability. To our knowledge, this study for the first time investigated the synergistic effects of MF-438 and 5-FU.

There is a need for more in vitro and in vivo studies to further validate the value of combination-based strategies as a beneficial CRC treatment. Confirmation of our results in other CRC cell lines and in animal models would strengthen the grounds for therapies based on lipid metabolism inhibition and antiproliferative synergy with 5-FU.

## 4. Materials and Methods

Methods and materials used in this experiment, to a large extent, have been described in our previous work [24]; thus, the main points of procedures are described briefly.

### 4.1. Tissue Samples

The study included tissue samples from 15 patients. Patients presented with locally advanced cancer at stage I or stage II and with regionally advanced malignancies with metastases to regional lymph nodes (stage III). None of the patients received preoperative neoadjuvant treatment. Tissue samples were collected from both the tumor and normal large intestinal mucosa at least 5 cm from the tumor interface. Each sample was divided into two parts: one was used for molecular analysis and the other was used for the preparation of routine hematoxylin and eosin (H&E)-stained microscopy slides for histopathological examination. The material for the molecular studies was frozen in liquid nitrogen immediately after collection and stored in aliquots at −80 °C until analysis. The protocol of the study was compliant with the Declaration of Helsinki of the World Medical Association and was granted approval from the Local Bioethics Committee at the Medical University of Gdansk (decision no. NKBN/487/2015). Written informed consent was obtained from all the subjects prior to the study.

### 4.2. Cell Culture

The human colon adenocarcinoma (HT-29) and normal human colon tissue (CCD 841 CoN) cell lines were obtained from the American Type Culture Collection (Manassas, VA, USA). Both cell lines were cultured in the supplier’s recommended basic medium supplemented with 10% fetal bovine serum, 100 units/mL penicillin, and 100 µg/mL streptomycin in an atmosphere of 5% CO_2_ at 37 °C. For gene expression analysis, both cell lines were seeded on 100 mm dishes at a seeding density of 2.2 × 10^6^. For the cell viability test, the HT-29 cell line was seeded on a 6-well culture plate at a seeding density of 0.3 × 10^6^.

### 4.3. Treatment with Experimental Agents

The following inhibitors were selected for the experiment: Avasimibe (ACAT1 inhibitor), Lovastatin (HMGCR inhibitor), and MF-438 (SCD1 inhibitor). 5-fluorouracil (5-FU) was selected as a representative of the chemotherapeutic drug widely used to treat patients with CRC. All experimental agents were dissolved in dimethyl sulfoxide (DMSO, Sigma-Aldrich, St. Louis, MO, USA) to create stock solutions. Experimental concentration ranges of each agent were selected based on current literature. In the first part of the experiment, each agent was tested separately to determine the IC50 dose. Twenty-four hours after HT-29 cell seeding, the basic medium was switched to either (1) fresh basic medium (control conditions), (2) medium with DMSO (solvent control), or (3) medium supplemented with different concentrations of tested agent. Solvent control contained such an amount of DMSO as the highest concentration used with experimental agents. In the second part, each inhibitor was paired with 5-FU, starting with an IC50 dose. Then the concentration of each was reduced until the lowest concentration at which IC50 was maintained. Cells were incubated with experimental agents for 72 h.

### 4.4. Cell Imaging and Viability Analysis

Images of cultured cells were acquired with the EVOS XL Core Cell Imaging System (Thermo Fisher Scientific, Waltham, MA, USA). Trypan Blue Solution was used as a cell stain to assess cell viability using the dye exclusion test. Cells were detached from the culture plate using trypsin and mixed with trypan blue solution according to the manufacturer’s instructions. The number of cells was determined using the Countess II Automated Cell Counter (Life Technologies, Carlsbad, CA, USA).

### 4.5. RNA Extraction and mRNA Level Analysis

The total RNA was isolated from frozen CRC patients’ tissues, HT-29, and CCD 841 CoN cells with RNeasy Plus Universal Kits (Qiagen, Hilden, Germany). RNA was reverse transcribed using recombinant Moloney murine leukemia virus reverse transcriptase and random hexamer primers (RevertAid First Strand cDNA Synthesis, Thermo Fisher Scientific) according to the manufacturer’s protocol. Duplicates of each sample were assayed via relative quantitative polymerase chain reaction using a CFX Connect Real-Time System (Bio-Rad, Hercules, CA, USA). β-Actin was used as the reference gene. Melting analysis was carried out at the end of the amplification cycle to verify the nonspecific amplification. The fold difference was calculated using the 2−ΔΔCt method.

### 4.6. Statistical Analysis

Statistical analyses were performed in GraphPad Prism 8 (GraphPad Software Inc., La Jolla, CA, USA). All results are shown as the mean ± SEM of at least three independent experiments. For more than two groups for comparison, statistical significance was examined by one-way analysis of variance (ANOVA) with Dunnett and Tukey’s HSD post hoc test. For gene expression assessment, a two-tailed Student *t*-test was used. Statistical significance was set as follows: * *p* ≤ 0.05; ** *p* ≤ 0.01; *** *p* ≤ 0.001. The agent’s combination effect was calculated using the SynergyFinder + web application. Results are shown on the gradient map. The HSA model was performed as the most representative of our data. The HSA model is one of the simplest reference models, which states that the expected combination effect is the maximum of the single drug responses at corresponding concentrations. Thus, the HSA synergy score, SHSA, is defined as SHSA = EA, B, …, N–max(EA, EB, …, EN), where EA, B, …, N is the combination effect between N drugs and EA, EB, …, EN are the measured responses of the single drugs [45]. The HSA synergy score specifically quantifies how the combination of two drugs performs compared to the best single drug in the combination. A score greater than 0 indicates that the combination is synergistic. The mean HSA synergy score summarizes the synergy scores obtained from multiple tests or experiments for a particular drug combination. Figures with schemes were created with BioRender.com.

## 5. Conclusions

In summary, our findings established that targeting lipid metabolism in combination with standard chemotherapeutics is a promising strategy that reduces the viability of CRC cells and allows for effective treatment with a lower drug dose. The most promising strategy among those used in our study appears to be the combination of chemotherapy along inhibition of cholesterol storage. The combination of 5-FU and Avasimibe has the greatest therapeutic potential among studied compounds.

## Data Availability

All study data can be viewed in this manuscript.

## Supplementary Materials

The following supporting information can be downloaded at: https://www.mdpi.com/article/10.3390/ijms26031186/s1.
