# Supplementary material for: Lipids Metabolism Inhibition Antiproliferative Synergy with 5-Fluorouracil in Human Colorectal Cancer Model
Source: Int J Mol Sci. 2025 Jan 30;26(3):1186. doi: 10.3390/ijms26031186 (PMC11818398; doi:10.3390/ijms26031186)
Supplement: Supplementary file 1 [file ijms-26-01186-s001.zip › ijms-3398743-supplementary.pdf]

Article - Supplementary materials

Lipids Metabolism Inhibition Antiproliferative Synergy with 5-Fluorouracil in Human Colorectal Cancer Model

Judyta Zabielska [1], Ewa Stelmanska [1], Sylwia Szrok-Jurga [1], Jarosław Kobiela [2] and Aleksandra Czumaj [3,*]

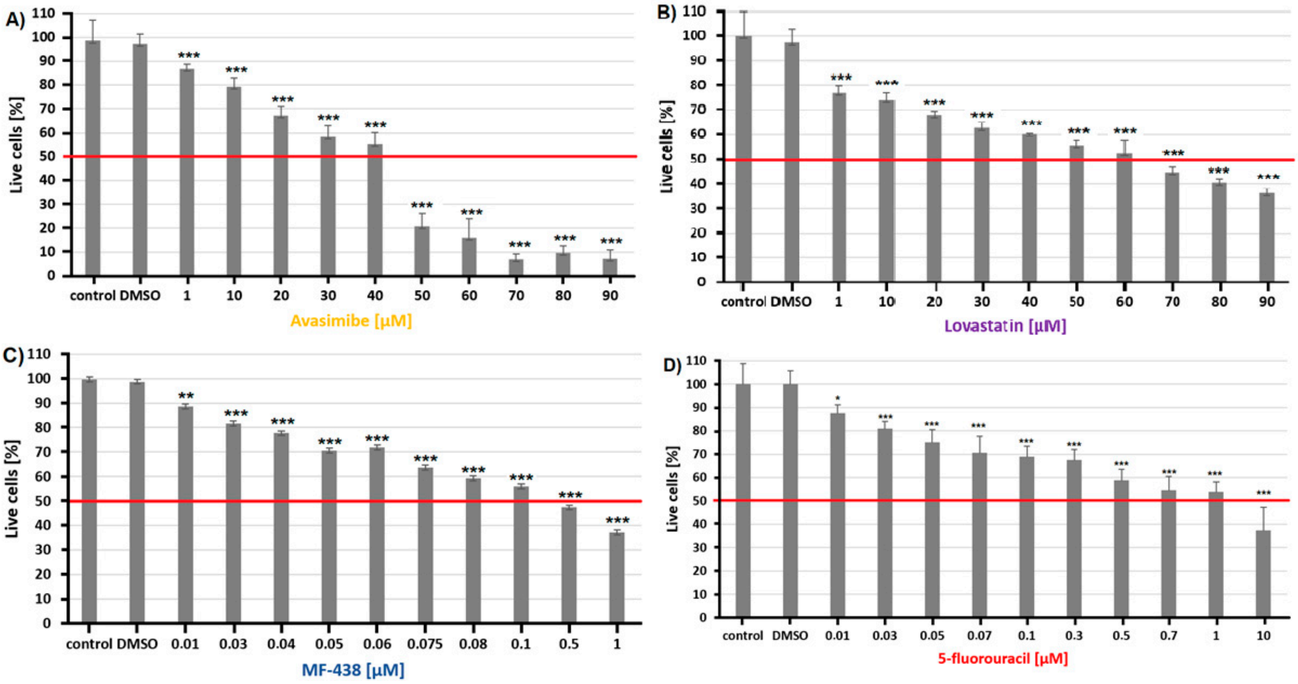

Supplementary Figure 1. The effect of treatment with increasing concentrations of (A) Avasimibe, (B) Lovastatin, (C) MF-438, and (D) 5-fluorouracil (µM) on cell viability of CCD-841-CoN control colon cell line. The red line represents the IC50 cut-off. Data are shown as mean ±SEM. Statistical significance in comparison to the control group is marked as follows: * for $p \le 0.05$; ** $p \le 0.01$;*** $p \le 0.001$.

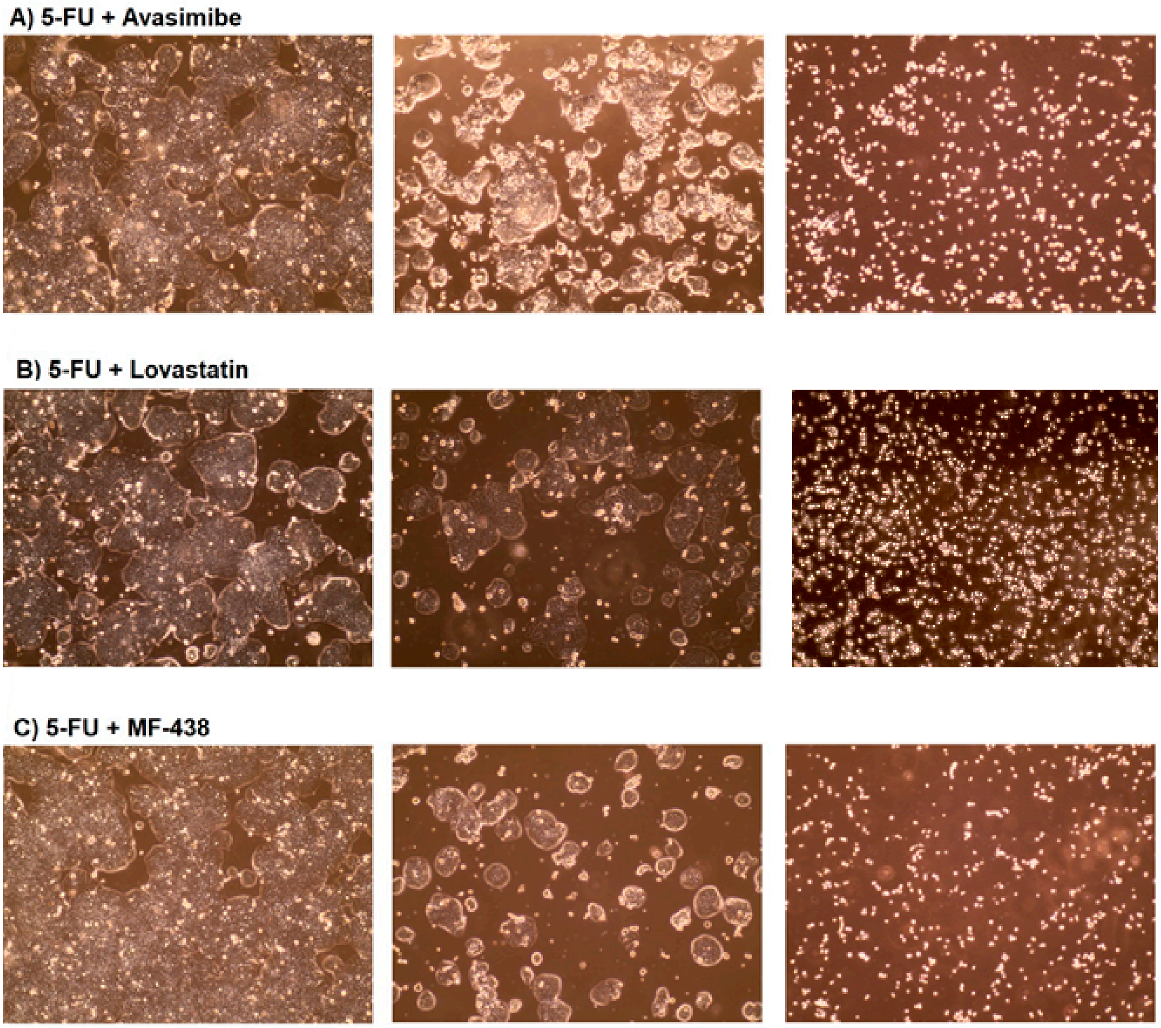

Supplementary Figure 2. Cell morphology after treatment with (A) 5-FU + Avasimibe, (B) 5-FU + Lovastatin, (C) 5-FU + MF-438. Random representations of control conditions, approximately IC50 and the highest tested concentration were selected.

Supplementary Table 1. Combination Index (CI) for selected combinations of 5-FU and lipid metabolism inhibitors. Selected combinations correspond with the bars on graphs 6a, 6c, and 6e. Calculation were conducted with CompuSyn software.

Concentration of 5-FU (μM)	Concentration of lipid inhibitor (μM)	CI
5-FU + Avasimibe		
0.05	5.0	0.24
0.1	10.0	0.22
0.15	15.0	0.21
0.2	20.0	0.098
0.25	25.0	0.0059
0.3	30.0	0.0018
5-FU + Lovastatin		
0.03	10.0	2.02
0.05	30.0	1.67
0.1	35.0	2.22
0.15	40.0	2.51
0.2	45.0	2.69
0.3	55.0	4.01
5-FU + MF-438		
0.05	0.035	29.63
0.1	0.040	5.32
0.15	0.045	0.43
0.2	0.05	0.24
0.25	0.055	0.065
0.3	0.06	0.040
